# Complete Sequencing of the Mitochondrial Genome of Opisthorchis felineus, Causative Agent of Opisthorchiasis

**Published:** 2009-04

**Authors:** V.A. Mordvinov, А.V. Mardanov, N.V. Ravin, S.V. Shekhovtsov, S.А. Demakov, А.V. Katokhin, N.А. Kolchanov, K.G. Skryabin

**Affiliations:** 1 Institute of Cytology and Genetics, Siberian Branch of Russian Academy of Sciences, 10 Lavrentieva Ave, Novosibirsk, 630090, Russia;; 2 Bioengineering Center, Russian Academy of Sciences, 7/1 60-letiya Oktyabrya Ave, Moscow, 117312, Russia

## Abstract

Opisthorchis felineus, a hepatic trematode, is the causative agent of opisthorchiasis, a dangerous disease in both human beings and animals. Opisthorchiasis is widespread in Russia, especially Western Siberia. The purpose of the present study was to determine the complete mitochondrial DNA sequence of this flatworm. Two parallel methods were employed: (1) capillary electrophoresis to sequence the mitochondrial genome fragments obtained through specific PCR amplification, and (2) high throughput sequencing of the DNA sample. Both methods made possible the determination of the complete nucleotide sequence of the O. felineus mitochondrial genome. The genome consists of a ring molecule 14,277 nt in length that contains 35 genes coding 2 rRNA, 22 tRNA, and 12 proteins: 3 subunits of cytochrome-C-oxidase, 7 subunits of NADH-dehydrogenase, B apocytochrome, and subunit 6 of ATP-synthetase.

Like many other flatworms, O. felineus is characterized by the absence of the ATP-synthetase subunit 8 gene. Nineteen out of the 22 tRNAs have a typical "clover leaf" structure. The tRNA(AGC) and tRNA-Cys genes lack DHU-loops, while the tRNA-Ser(UCA) has 2 alternative structures: one with a DHU-loop, and one without it. Analyzing the results obtained from the high throughput sequencing revealed 45 single-nucleotide polymorphisms within the mitochondrial genome. The results obtained in this study may be used in the development of molecular diagnostic methods for opisthorchiasis. This study shows that high throughput sequencing is a fast and effective method for decoding the mitochondrial genome of animals.

## INTRODUCTION

The flatworm Opisthorchis felineus (class: Trematoda, family: Opisthorchiidae) is a parasitic liver fluke in both human beings and animals. An estimated 2 million people worldwide are infected with opisthorchiasis, most of them in Russia and countries of the former Soviet block, such as Ukraine, Belarus and Kazakhstan [1, 2]. Within some of the northern settlements in these regions, up to 90% of the population is infected with opisthorchiasis [1].

Although O. felineus has been studied for over a century, the lack of knowledge about its specific indentifying characteristics has meant that many questions about its prevalence and about how it evolves remain to be answered. Previous molecular analyses of these flukes have not provided molecular markers specific enough to be effective for the purposes of present-day studies [3, 4, 5], but the complete decoding of this trematode's mitochondrial genome may enable specific and effective molecular markers to be created, which would have far-ranging applications in research.

The mitochondrial DNA (mtDNA) of most species of animals has some unique features, such as its maternal pattern of inheritance, the absence of recombination and its higher replication rate, which distinguish it from nuclear DNA [6], and which make it a potentially unequalled tool for identification in phylogenetic and phylogeographic studies.

The number of sequenced genomes continues to increase, and now they are widely used for selecting genetic markers characterized by a high evolution rate, and for creating high-resolution phylogenetic trees in which both the sequences proper and the individual gene sequences can be used as markers. 

Two of the methods available for the complete sequencing and structural analysis of the mitochondrial genome of O. felineus are the subject of the present review. 

## MATERIALS AND METHODS

### Biomaterial source and DNA recovery

The O. felineus samples were recovered from an infected cat from the Ust-Tula settlement (Novosibirsk Region, Russia). The morphological features allowed specialists from the Parasitology and Ichthyology Laboratory (Institute of Systematic and Ecology of Animals, Russian Academy of Sciences) to determine the species. Both sequencing procedures involved the recovery of DNA from the pooled samples using the phenol-chloroform method [7].

### Decoding of the O. felineus mtDNA sequence using capillary electrophoresis, after P. Senger

The conserved sequences characteristic of the trematode genomes were identified by comparing the mitochondrial genomes of the Fasciola hepatica (AF216697), Paragonimus westermani (AF216698) and Schistosoma mansoni (AF216698) trematodes using the MEME/MAST programs (http://meme.sdsc.edu/). Universal primers were selected on the basis of those sequences, as well as on the basis of such published sequences as Clonorchis sinensis (DQ116944, AY264851) and O. viverrini (DQ882172, DQ119551). These primers helped to create a set of amplicons, approximately 1,000 pn long, whose sequences were then used to synthesize new primers. Then, the remaining overlapping fragments of the mitogenome were amplified. Most amplicons were directly sequenced; some amplicons were cloned, and then at least three clones were subjected to sequencing. The mtDNA sequencing was performed using the Applied Biosystems ABI PRISM 3100 Avant Genetic Analyzer in the DNA Sequencing Institute, Siberian Branch of the Russian Academy of Sciences. The complete sequence of O. felineus mtDNA can be found in the GenBank (NC_011127).

### Decoding of the O. felineus mtDNA sequence using the high throughput sequencing method

In order to determine the O. felineus mtDNA sequence using the high throughput sequencing method, we employed the techniques developed by the 454 Life Science Company with the GS FLX genome analyzer. Having obtained the library of random DNA fragments, we carried out the clonal amplification of the DNA molecules related to the microparticles in the water-in-oil emulsions, as well as the sequencing with the GS FLX genome analyzer using a reagents kit and following the protocols established by the Roche Laboratory. One run of the device (12 hours) allowed us to determine 100 mln. nt; the average length of "reading" was about 220 nt. 

The set of overlapping sequences obtained using the GS FLX genome analyzer was then assembled into contiguous clones using the GS de novo Assembler program pack (Roche Diagnostics, Roche Applied Science). Finally, the complete nucleotide sequence of the contiguous clone was determined to be mitochondrial genome, 14, 277 nt in length. The average mtDNA reading frequency was 30. 

### Analysis of Bioinformation

The analysis both of the sequences and of the assembled genome was performed with the Vector NTI 7 program (Informax Inc.). Similar sequences were searched for in the GenBank's biological sequences databases (http://www.ncbi.nlm.nih.gov/blast). The flatworm's mitochondrial genetic code was used to translate protein-coding sequences [8]. Most tRNA were detected by the tRNAscan-SE program, [9] while secondary structures of other flatworms were found manually. 

In order to identify potential single nucleotide polymorphisms (SNP), some sequences determined during the course of sequencing were aligned relative to the "consensus" sequence of O. felineus mtDNA using the GS reference mapper program (Roche). SNPs were detected during the course of at least three individual readings at those points where their sequences did not coincide with the "consensus" sequence. All points where the complete mtDNA sequences determined by capillary electrophoresis and high throughput sequencing methods were not consistent were referred to as SNP as well.

## RESULTS AND DISCUSSION

### Methods of mtDNA sequencing 

Due to their relatively short lengths, animal mitochondrial genomes were among the first objects of genomic investigation [10], and to date, hundreds of mtDNA sequences are known.

The standard method for decoding the mitochondrial genome involves the recovery of mitochondria from the cells and the creation of a mitochondrial DNA sample maximally purified of genome DNA. The following Sanger sequencing suggests the genome decomposition into randomly chosen fragments, cloning using the plasmid vector (library of random fragments), and sequencing of the clones produced using capillary electrophoresis. Since the fragments are overlapping, the sequences produced may be combined into a complete mtDNA sequence. In the present study, for the specific recovery of mitochondrial sequences, we used data on the mtDNA structure of closely related helminthes, which allowed us to identify the conserved sites of the genome, and to amplify the O. felineus mtDNA fragments occurring between them using the PCR method. The sequences of the fragments obtained were determined by capillary electrophoresis and were combined into a complete mtDNA sequence 14,277 nt in length. 

A new method, which makes it possible to detect the genome sequences de novo, is the high throughput sequencing method [11], developed by the 454 Life Science Company using the GS FLX genome analyzer. This method involves the fragmentation of up to 300-800 nt of DNA, the amplification of the individual DNA fragments related to microparticles in microdrops formed in the water-in-oil emulsions, the injection of nanoparticles containing immobilized amplified fragments into the microcells on the glass sheet, parallel high throughput sequencing, and the registration of the results obtained from each of the few hundred thousand cells on the glass sheet. The average reading length is approximately 200 nt, and one run of the device can analyze a sequence up to 100 mln nt in length. The large volume of sequences detected using this method allowed us to reject the specific recovery of the mitochondrial genome fragments and to use the sample of "total" O. felineus genome DNA for the sequencing. In spite of the fact that the share of the mtDNA sequences was less that 1% of the whole sequencing volume, it was enough for the reading of mtDNA with 30-fold overlapping that provided a complete "assembly" of the mitochondrial genome sequence following only one run of the GS FLX genome analyzer.

### Major characteristics of the O. felineus mitochondrial genome 

The O. felineus mitochondrial genome is a ring molecule, 14,277 nt in length. It is the shortest among the currently known mitochondrial genomes of trematodes [12]. Analysis of the genome sequence confirmed the presence of typical mitochondrial genes: 12 protein-coding genes (ATP-synthetase subunit 8 is absent), 22 tRNA-, and 2 rRNA-coding genes (Table 1).

**Table 1:** Gene pattern of the O. felineus mitochondrial genome.

Gene	Length, pn	Start-codon	Stop-codon	Gene	Length, pn	Start-codon	Stop-codon
cox3	642	ATG	TAG	nd3	354	GTG	TAG
tRNA-His	67			tRNA-Ser(AGN)	61		
cob	1110	ATG	TAG	tRNA-Trp	68		
nd4L	261	ATG	TAG	cox1	1560	GTG	TAG
nd4	1275	ATG	TAG	tRNA-Thr	63		
tRNA-Gln	63			16S rRNA	994		
tRNA-Phe	66			tRNA-Cys	60		
tRNA-Met	68			12S rRNA	780		
atp6	513	ATG	TAG	cox2	639	ATG	TAG
nd2	867	ATG	TAG	nd6	459	ATG	TAG
tRNA-Val	65			tRNA-Tyr	62		
tRNA-Ala	62			tRNA-Leu(CUN)	64		
tRNA-Asp	67			tRNA-Ser(UCN)	72		
nd1	900	GTG	TAG	tRNA-Leu(UUR)	65		
tRNA-Asn	71			tRNA-Arg	68		
tRNA-Pro	64			nd5	1602	ATG	TAG
tRNA-Ile	62			tRNA-Glu	72		
tRNA-Lys	65			tRNA-Gly	67		

**Fig. 1.**
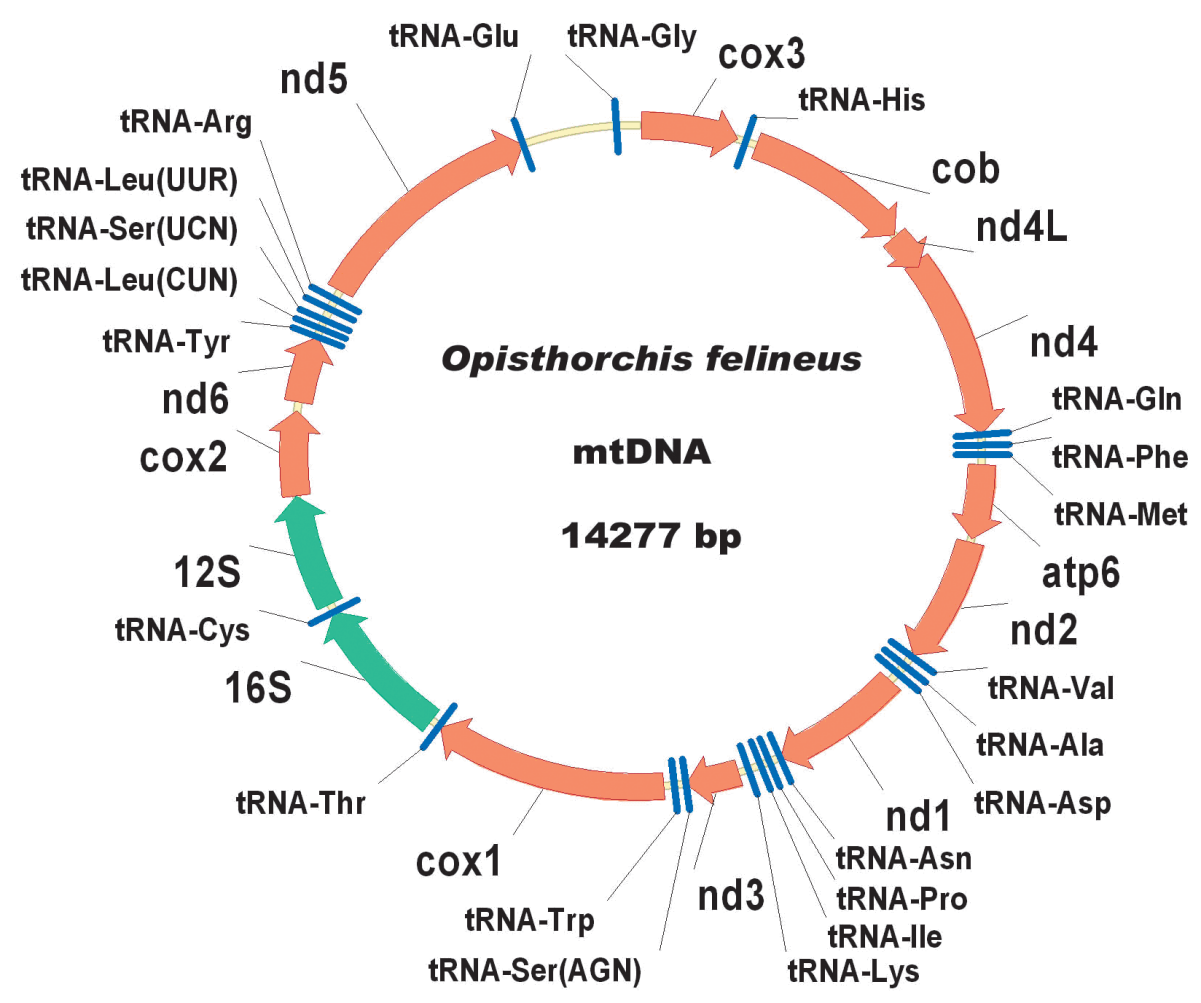
Genetic map of the O. felineus mitochondrial genome.

As with other flatworms, all genes are transcribed from one chain (Fig. 1). The gene sequence of the O. felineus mitochondrial genome is similar to that of F. hepatica [13]; 40 pn of nd4L and nd4 genes are overlapped for different reading frames. 

All well-known flatworm mitochondrial genomes, except for the P. westermani genome, are А/Т-rich. The O. felineus mitochondrial genome contains 60% А+Т; moreover, the coding strand is rich in thimine (43%) compared to adenine (17%), guanine (28%), and cytosine (12%). The nucleotide composition is variable in different parts of the O. felineus genome, especially in the third position of codons of protein-coding genes, where the cytosine content is only 8%. Codons ending in T and G are more frequent than those ending in A and C. The most frequently appearing codons are ТТТ, GTT, and TTG. The percentage of ТТТ codons represents almost 10% of the total number, while all codons composed of A and C account for only 2% (Table 2). As with other trematode mitochondrial genomes, the start-codons are ATG and GTG, while the stop-codon is TAG. The TGA codon codes for tryptophan, while TAA is not used at all. Truncated stop-codons were not found in the O. felineus mitochondrial genome (Table 1). 

**Table 2:** Frequency of occurrence of different codons in the O. felineus mitochondrial genome.

UUU	Phe	334	9.9 %	UCU	Ser	107	3.2 %	UAU	Tyr	146	4.3 %	UGU	Cys	89	2.6 %
UUC	Phe	40	1.2 %	UCC	Ser	22	0.7 %	UAC	Tyr	17	0.5 %	UGC	Cys	15	0.4 %
UUA	Leu	125	3.7 %	UCA	Ser	26	0.8 %	UAA	-	0	0 %	UGA	Trp	33	1.0 %
UUG	Leu	236	7.0 %	UCG	Ser	36	1.1 %	UAG	stop	12	0.4 %	UGG	Trp	80	2.4 %
CUU	Leu	107	3.2 %	CCU	Pro	43	1.3 %	CAU	His	49	1.5 %	CGU	Arg	47	1.4 %
CUC	Leu	14	0.4 %	CCC	Pro	19	0.6 %	CAC	His	7	0.2 %	CGC	Arg	4	0.1 %
CUA	Leu	26	0.8 %	CCA	Pro	7	0.2 %	CAA	Gln	12	0.4 %	CGA	Arg	3	0.1 %
CUG	Leu	41	1.2 %	CCG	Pro	21	0.6 %	CAG	Gln	21	0.6 %	CGG	Arg	23	0.7 %
AUU	Ile	103	3.1 %	ACU	Thr	60	1.8 %	AAU	Asn	44	1.3 %	AGU	Ser	72	2.1 %
AUC	Ile	18	0.5 %	ACC	Thr	11	0.3 %	AAC	Asn	7	0.2 %	AGC	Ser	15	0.4 %
AUA	Met	55	1.6 %	ACA	Thr	8	0.2 %	AAA	Asn	17	0.5 %	AGA	Ser	15	0.4 %
AUG	Met	101	3.0 %	ACG	Thr	16	0.5 %	AAG	Lys	44	1.3 %	AGG	Ser	47	1.4 %
GUU	Val	215	6.4 %	GCU	Ala	21	0.6 %	GAU	Asp	69	2.0 %	GGU	Gly	133	3.9 %
GUC	Val	15	0.4 %	GCC	Ala	76	2.3 %	GAC	Asp	3	0.1 %	GGC	Gly	35	1.0 %
GUA	Val	39	1.2 %	GCA	Ala	5	0.2 %	GAA	Glu	6	0.2 %	GGA	Gly	28	0.8 %
GUG	Val	138	4.1 %	GCG	Ala	36	1.1 %	GAG	Glu	71	2.1 %	GGG	Gly	105	3.1 %

The corresponding amino-acid and the frequency of occurrence in the mtDNA genes are indicated for each codon. Differences from the standard genetic code are underlined.

**Fig. 2.**
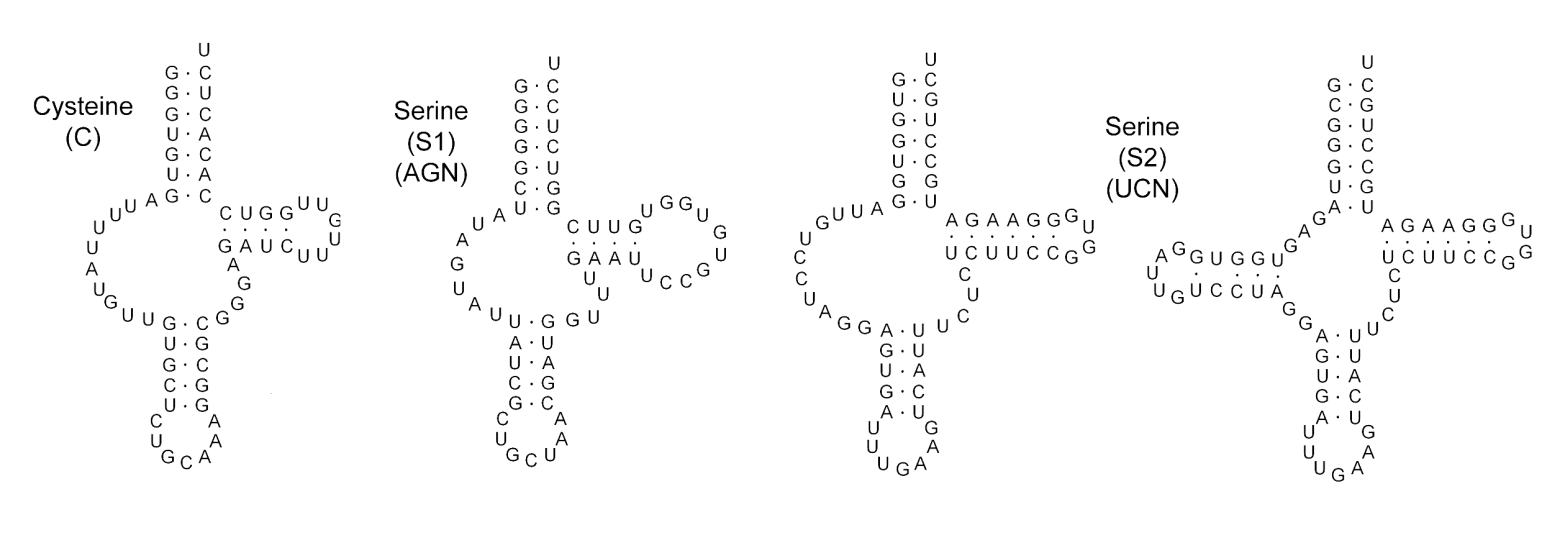
Secondary structures of tRNA: tRNA-Ser(AGN), tRNA-Cys, and two possible alternative structures of tRNA-Ser(UCN).

The length of tRNA genes in the O. felineus mitochondrial genome ranges from 59 to 72 nucleotides. Most tRNA genes are combined in clusters composed of up to five genes. Nineteen out of 22 tRNA genes are characterized by the typical "clover leaf" structure. As in all trematodes, tRNA-Ser(AGN) is lacking in the DHU-loop. The tRNA-Cys, as in some schistosomes, does not have the DHU-loop [14]. The tRNA-Ser(UCN) gene can have two alternative structures: one with the DHU-loop and one without it (Fig. 2). 

In addition to short intervals between consecutive genes, flatworm genomes often have long non-coding regions, which are believed to be sequences necessary for the initiation of the mtDNA replication and transcription. As in the F. hepatica genome, the O. felineus non-coding region located between the tRNA-Glu and cox3 genes is divided into 2 parts by the tRNA-Gly gene. In contrast to the non-coding regions of the mitochondrial genomes of other flatworms, the O. felineus non-coding regions contain neither long tandem repeats, nor sequences able to form long hairpin structures. 

An open reading frame, 402 pn in length, was detected in the O. felineus non-coding region. A search for similar sequences within the database of biological sequences using both the nucleotide and the amino-acid sequence did not yield any results. Quite long open reading frames different from well-know proteins were also found in the non-coding regions of the mtDNA of other flatworm species: F. hepatica, cestodes Hymenolepis diminuta [15], and monogeneas Microcotyle sebastis [16]. These reading frames likely code for functional proteins; however, this hypothesis needs to be investigated further in future studies. 

The mtDNA non-coding region may be used to develop a molecular method for the specific identification of O. felineus. The homology levels between the O. felineus mtDNA sequences and that of two related trematodes, C. sinensis (FJ381664) and F. hepatica (AF216697), from which the mitochondrial genome sequences are well-known, amount to 78% and 64%, respectively. However, these three sequences of non-coding regions located between the tRNA-Glu and cox3 genes do not have significant homology either between themselves or with other sequences contained in the GenBank. 

### Single nucleotide polymorphisms in the O. felineus mtDNA 

In the course of the mtDNA sequencing using the high throughput sequencing method, each nucleotide in the genome was "read" an average of 30 times in the process of sequencing of the clonal-amplified individual fragments of the O. felineus mtDNA molecules. 

Comparison of the sequences obtained during the course of individual reading with the consensus sequence permitted the identification of single nucleotide polymorphisms (SNP), which are present in different mtDNA molecules in one organism. Since both Sanger sequencing and high throughput sequencing were performed with DNA recovered from several O. felineus species, comparing the corresponding mtDNA sequences makes it possible to estimate the frequency of hyplotypes occurrence in each SNP. 

Data from 45 detected SNPs is presented in Table 3. Most SNPs in both animal and human mtDNA [17] involve Т:С and A:G substitutions (corresponding to T:C on the lower strand), which do not cause an amino-acid substitution in the protein products of the corresponding genes. It should be noted that some SNPs looked specific for the mtDNA sequence decoded by one of two technologies and were not found (or were only rarely found) in other sequences. The difference in allele frequency is likely to be the result of errors specific to the PCR-based methods for the amplification and sequencing of the heterogeneous amplicon mixture, while the ratio of SNP alleles obtained in the process of sequencing individual fragments must be extremely precise. 

**Table 3:** Single nucleotide polymorphisms detected in the O. felineus mitogenome.

№	Number of occurrences in mtDNA and allele determined by Sanger sequencing	Allele frequencies in sequences determined duringt the course of high throughput se-quencing	Gene	Replaceable codon	Amino-acid re-placement
1	361	T	C-3 / T-27	cox3	tgg/cgg	W/R
2	1068	C	T-3 / C-25	cytB	tac/tat	-
3	1195	C	T-8 / C-17	cytB	cta/tta	-
4	1300	C	T-4 / C-23	cytB	ctg/ttg	-
5	1524	A	A-1 / G-28	cytB	caa/cag	-
6	1599	T	C-5 / T-28	cytB	ctg/ccg	L/P
7	1860	C	C-1 / T-24	cytB	ggc/ggt	-
8	1899	C	T-4 / C-24	nd4L	cct/tct	P/S
9	2025	T	C-4 / T-28	nd4L	tta/cta	-
10	2034	C	T-4 / C-28	nd4L	cgg/tgg	R/W
11	2039	C	T-4 / C-28	nd4L	ggc/ggt	-
12	3104	T	C-5 / T-19	nd4	ttg/ctg	-
13	3228	C	T-3 / C-25	nd4	gcg/gtg	A/V
14	3245	T	C-3 / T-27	nd4	tta/cta	-
15	3260	C	T-3 / C-26	nd4	ctg/ttg	-
16	3674	A	G-6 / A-28	atp6	aat/agt	N/S
17	3827	G	G-0 / A-39	atp6	ggt/gat	G/D
18	3915	A	A-0 / G-34	atp6	cta/ctg	-
19	3921	T	C-6 / T-28	atp6	tat/tac	-
20	3935	T	T-0 / C-35	atp6	gtg/gcg	V/A
21	4507	A	A-1 / G-20	nd2	ata/gta	I/V
22	4548	C	T-11 / C-14	nd2	agc/agt	-
23	4707	T	T-0 / C-33	nd2	tct/tcc	-
24	4710	T	T-0 / G-33	nd2	cct/ccg	-
25	5390	T	T-0 / A-36	nd1	aat/aaa	N/K
26	5684	C	C-2 / T-21	nd1	gcc/gct	-
27	6158	C	T-5 / C-32	-		
28	6175	C	T-5 / C-33	tRNA-Asn		
29	6314	T	C-3 / T-24	tRNA-Ile		
30	6650	A	A-2 / G-30	nad3	gta/gtg	-
31	6810	C	T-4 / C-30	-		
32	7865	A	A-0 / G-31	cox1	tca/tcg	-
33	8669	A	G-4 / A-27	16S rRNA		
34	10692	T	C-3 / T-17	cox2	ata/aca	I/T
35	11152	A	G-8 / A-23	nd6	cca/ccg	-
36	11880	C	T-4 / C-36	tRNA-Arg		
37	12186	G	G-1 / C-25	nd5	gtt/ctt	V/L
38	12326	T	A-3 / T-24	nd5	cgt/cga	-
39	12533	G	A-5 / G-22	nd5	gtg/gta	-
40	13403	G	A-4 / G-23	nd5	tcg/tca	-
41	13589	G	G-0 / A-11	-		
42	13993	C	C-0 / T-30	-		
43	14001	G	G-0 / A-18	-		
44	14007	C	C-0 / T-8	-		
45	14212	C	C-0 / T-9	-		

In the future, the data on specific SNPs and their frequency of occurrence in mtDNA may be used as molecular markers in studies of the natural populations of O. felineus, as well as in the analysis of the pathogenic pathways of this trematode in human populations.

## CONCLUSION

This review contains the results of the complete sequencing of the O. felineus flatworm mtDNA obtained using two methods. The first method involved the amplification and sequencing of the mtDNA using capillary electrophoresis. Parallel high throughput sequencing of the animal genome DNA sample is performed without any preliminary enrichment with the mtDNA sequences. This enables the complete de-novo sequencing of the mitochondrial genome. The high throughput sequencing method using the GS FLX genome analyzer may be used for the rapid decoding of animal mitochondrial genomes and for the identification of polymorphisms. The newly generated data on the nucleotide sequence of the O. felineus mitochondrial genome may be utilized in the development of specific molecular diagnostic methods for opisthorchiasis. 

## Acknowledgements

The work was supported by the program "Genomics, Proteomics, and Bioinformatics" of the Institute of Cytology and Genetics, Russian Academy of Sciences, and by the Federal Agency for Science and Innovations (project no. 02.552.11.7045) of the Bioengineering Center, Russian Academy of Sciences. We would like to thank N.I. Yurlova and K.P. Fedorov, members of the Institute of Systematic and Ecology of Animals, Russian Academy of Sciences, for assistance in the identification of O. felineus. 
